# Dogs can infer implicit information from human emotional expressions

**DOI:** 10.1007/s10071-021-01544-x

**Published:** 2021-08-14

**Authors:** Natalia Albuquerque, Daniel S. Mills, Kun Guo, Anna Wilkinson, Briseida Resende

**Affiliations:** 1 Institute of Psychology, University of São Paulo, São Paulo, Brazil; 2 School of Life Sciences, University of Lincoln, Lincoln, UK; 3 School of Psychology, University of Lincoln, Lincoln, UK

**Keywords:** *Canis familiaris*, Decision-making, Emotions, Social behaviour, Social cognition

## Abstract

The ability to infer emotional states and their wider consequences requires the establishment of relationships between the emotional display and subsequent actions. These abilities, together with the use of emotional information from others in social decision making, are cognitively demanding and require inferential skills that extend beyond the immediate perception of the current behaviour of another individual. They may include predictions of the significance of the emotional states being expressed. These abilities were previously believed to be exclusive to primates. In this study, we presented adult domestic dogs with a social interaction between two unfamiliar people, which could be positive, negative or neutral. After passively witnessing the actors engaging silently with each other and with the environment, dogs were given the opportunity to approach a food resource that varied in accessibility. We found that the available emotional information was more relevant than the motivation of the actors (i.e. giving something or receiving something) in predicting the dogs’ responses. Thus, dogs were able to access implicit information from the actors’ emotional states and appropriately use the affective information to make context-dependent decisions. The findings demonstrate that a non-human animal can actively acquire information from emotional expressions, infer some form of emotional state and use this functionally to make decisions.

**Supplementary Information:**

The online version contains supplementary material available at 10.1007/s10071-021-01544-x.

## Introduction

Some animal species can use the behavioural changes of others to identify cues predictive of their motivations and intentions (Call et al. 2004), and associate emotional cues with specific outcomes to direct their behaviour (Buttelmann and Tomasello 2013; Buttelmann et al. 2009; Morimoto and Fujita 2012; Waller et al. 2016). However, it is unknown whether non-human individuals can infer the potential consequences of others’ emotional expressions to make decisions in ecologically relevant contexts. That is, whether animals are capable of retrieving information from their memory about their experience with certain emotional displays, the contexts in which they occur and their possible behavioural outcomes, and use this information to solve problems.

Group living allows animals to benefit from the knowledge of other individuals (e.g. social learning, use of public information) to optimise activities (e.g. Albuquerque et al. 2021; Bugnyar and Heinrich 2005; Eschar et al. 2016; Kendal et al. 2009). Anticipating others’ behaviour and adjusting one’s own behaviour according to environmental demands to obtain access to resources and increase fitness is cognitively challenging but highly advantageous, and so may affect how animals behave and make decisions (McFarland et al. 2013). Such abilities may affect what tactics individuals may use when engaging with their surroundings (Eschar et al. 2016). According to McFarland (1977), optimal decision-making involves the recognition and evaluation of the trade-offs associated with the consequences of making choices and the preferential use of certain information. Moreover, optimal decisions depend on the extent to which individuals have inferential skills that extend beyond the perception of the current behaviour of another individual and their ability to make predictions of the functional significance of emotional states. In adult humans, the capacity to interpret emotional expressions allows an observer to make inferences about subjective information relating to the producer of the signal (van Kleef 2009), such as understanding someone might be feeling happy and might be available for a friendly conversation by watching them smile i.e. functionally use of implicit information about the sender based on their emotional state. Children also have the ability to make inferences about the emotional states of others (Thompson 1987), however, the extent to which this occurs in non-human animal species remains unknown.

Dogs are excellent models to explore these questions due to their unique evolutionary history within complex emotionally-mediated heterospecific environments. Not only have they lived alongside humans for more than ten thousand years (Irving-Pease et al. 2018; Perri et al. 2021), but there is also increasing evidence of their ability to perceive (Correia-Caeiro et al. 2020), categorise (Müller et al. 2015), recognise (Albuquerque et al. 2016) and respond to human emotional expressions (Albuquerque et al. 2018). Moreover, there is a body of literature showing that dogs pay attention to third-party interactions and can obtain information from humans through passive observations (e.g. Chijiwa et al. 2015; Piotti et al. 2017). Here, we look at third-party social exchanges to investigate whether they are also able to obtain and functionally use implicit information from people, and whether the use of this information is different depending on human interaction. We used emotional information contained in social interactions, such as that related to overt visual expressions.

Visual exploration is fundamental in dog–human interactions. Gazing is a critical behaviour from dogs’ repertoire, serving not only to inspect others’ (e.g. Passalacqua et al. 2011; Somppi et al. 2016), but also to request help from humans (Cavalli et al. 2020). Dogs possess a relatively limited repertoire of affective behavioural expressions compared to humans (Caeiro et al. 2017) and yet seem to have remarkable interspecific communication abilities with people (Kaminski et al. 2013). We hypothesise that dogs’ domestication history and ontogenetic experience emphasises adaptive advantages associated with the ability to recognise and use—at a functional level—the implicit information contained in social exchanges, such as emotional expressions and states. To investigate whether emotional information can affect decision-making in a non-human animal, we analysed the behaviour of 91 domestic dogs in an ecologically relevant social task to test two main hypotheses. First, that dogs can infer the potential consequences associated with certain emotional states (i.e. what sort of behaviour might follow an emotional event), as evidenced by them making functional use of indirect heterospecific emotional cues. Second, that dogs do not use this information indiscriminately; rather, they take into account different elements of a social problem when using this information.

In our experiment, dogs witnessed silent social exchanges between two unfamiliar humans who displayed visual emotional expressions, triggered by and directed towards a neutral object either in a positively-valenced interaction (*n* = 27), a negatively-valenced interaction (*n* = 33) or a neutral interaction (*n* = 31). Dogs were then given a choice of whom to interact with to access a desired food resource. This occurred in one of two possible contexts: (a) they could reach the food by themselves (direct access, *n* = 43) or (b) they could see but not access the food without help from one of the humans (indirect access, *n* = 48).

We predicted that if dogs acquire and use affective information functionally then they should adjust their behaviour towards the person they perceive to be more positive in the indirect food condition. By contrast, such a differential behavioural response would be unnecessary when dogs can access food directly, if the dogs are using the information functionally, as they would be less dependent on the information provided by the actors for accessing the resource.

## Methods

### Subjects

We tested 114 (68 female and 46 male) healthy adult domestic pet dogs (*Canis familiaris*) of various breeds (see Table S1 for detailed information). All subjects had been living with their human family for at least six months and were used to interacting with new people and being in unfamiliar environments. From this sample, 91 (56 females and 35 males) dogs were included in the final dataset according to the following criteria. In the observation phase any dog that showed signs of stress, did not look at both actors, or did not watch the interaction for at least five seconds was excluded. This last criterion was selected as this time has been shown to be sufficient for dogs to acquire information for emotion processing (Albuquerque et al. 2016). Six dogs were excluded in this stage. The next stage considered any interference during testing, e.g. sudden noises from the outside, owner interacting with the dog in any way. Seventeen further dogs were excluded at this point.

Ethical approval was granted by the University of São Paulo ethics committee and all procedures complied with the ethical guidance for the use of animals by the International Society for Applied Ethology. We obtained signed informed consent from owners for all subjects. Dogs were monitored throughout the entire experimental session to ensure that they did not exhibit signs of stress. Dogs had their owners close to them at all times and were only handled by them.

### Stimulus

We used a controlled yet naturalistic setting where subjects had the opportunity to witness a social interaction between two completely unfamiliar humans, which involved differential indirect display of emotional expressions.

Dogs observed two female Caucasian actors of the same age who always wore the same clothing (within and between subjects), had their hair wrapped in a bun, wore no make-up nor jewellery and had no distinct marks visible on their faces or bodies. The actors underwent an extensive training period prior to the beginning of the study to ensure similarity in the expression of emotion and synchronisation of the movements (within and between dogs).

### Procedure

**Fig. 1:**
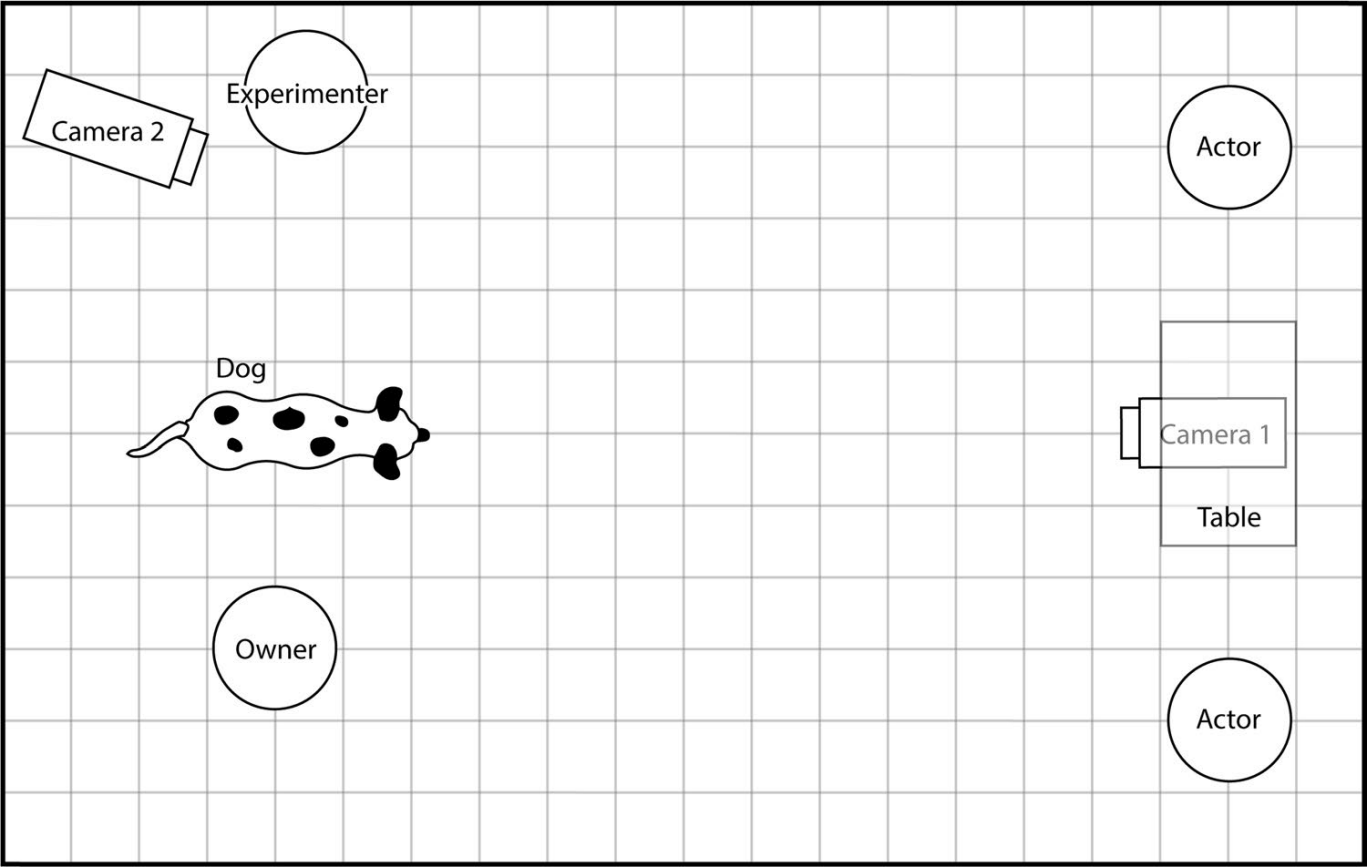
Schematic view of the experimental setting. The set up consisted of a table, two actors positioned next to it, the experimenter, the owner and the dog. Every owner stood in the same area of the room, which was marked on the floor and indicated to them prior to the experiment. The behaviour of the subjects was recorded with two digital video cameras (camera 1 and camera 2), which registered their responses from different angles

Each subject was tested in a single experimental session, which took place at the Institute of Psychology of the University of São Paulo (Brazil) in a rectangular experimental room (185 cm × 308 cm). All testing was done in the same inside space, which was marked for each object and person position and controlled for not varying between subjects, during the entire study. At one end of the room, there was a small table against the wall which had two bowls and a set of three identical black opaque discs (they were produced for the purpose of this experiment and the dogs had never seen, touched or interacted with them) on it. Two stools were placed one on each side of the table and a digital video camera was positioned below the table to give a clear frontal view of the dog (see Fig. 1). At the opposite end of the room, there were marks on the floor showing the positioning of the dog and the owner, the experimenter and another video camera.

Our experiment consisted of two phases, an observation phase and a response phase (see Fig. 1 for an overview of the experimental set up and the online supplement for additional experimental details).

#### Observation phase

The actors were initially standing still, quiet with neutral facial and body expression beside a table, upon which two bowls with desirable food were placed. We used small pieces of dried-meat treats to bait the bowls. The amount of treats, as well as the size of the pieces, varied according to the size of the animal, and was aimed at motivating the dog to approach the bowls. None of the dogs were food deprived. The only instruction provided to the owners was not to feed their dogs just before bringing them to the test setting.

During the observation phase, dogs were positioned two metres away from the actors/demonstrators. The interaction then proceeded as follows. One actor (giver) turned and picked up one of three identical opaque black discs placed on a table (see information about the experimental room below), handed it to the other actor (receiver) and returned to her initial position. The aim was to create a giver-receiver dynamic. This was used because it is a common and natural type of interaction between two people that dogs are used to watching and because it would allow us to tease apart whether the dogs were making their choices based on the motivation/role of the humans, or, based on the humans’ emotional state. The three black discs were chosen to guarantee intrinsically neutral objects.

The giver remained neutral throughout the entire session. The receiver turned to receive the disc from the giver, held it, looked at it and then reacted to it. The reaction could be positive (happy), negative (angry) or neutral. The receiver turned back to her initial position while holding and looking at the disc and exhibiting the designated emotional expression (Figure S1). Then, while continuing to react emotionally, she looked ahead, turned towards the table and placed the object back with the set of discs. The emotional reaction was completely silent. This was repeated three times, each with a different disc.

The entire interaction lasted 30 s (Videos S1–S3). We used silent displays to control for variation in the demonstration of the interaction between subjects, which can easily occur in a naturalistic setting when using vocalisations or emotionally charged sounds that are not pre-recorded. Pre-recorded sounds suffer from a loss in natural information and sonority, possibly disrupting acoustic perception and was therefore not used (Fukuzawa et al. 2005). The entire demonstration was inspired by real life social interactions between two human beings, which can be extremely nuanced across contexts and can occur with and without the display of emotions (see videos S1–S3).

After the third exchange, each actor sat on a stool and remained looking down (reading a paper) with a neutral body posture and facial expression. Upon sitting, the situation could proceed in two distinct ways: (i) each actor held a baited bowl next to their body and made the food directly accessible to the dogs (direct access) or (ii) the actors left the baited bowls on the table. These were stacked one on top of the other thus creating what was apparently a “single” bowl. The food remained unreachable (indirect access). Position of each actor (left or right), role of each actor (giving or receiving), emotional display (positive, negative or neutral) and food accessibility (direct or indirect access to the food) was randomised between subjects.

#### Response phase

Once the interactions between actors were completed, the response phase started. During the response phase, the dogs were released and their spontaneous behaviour was recorded for 30 s (videos S1–S3). We coded dogs’ choices along with visual exploration, body orientation, position in the room and sniffing behaviour.

Actors never looked at, nor interacted with, the dogs in either phase of the experiment. Each dog was tested only once to control for habituation and learning effects and ensure independence of the data. Of the analysed sample, 27 dogs were tested in the positive condition (happy receiver, neutral giver), 33 in the negative conditions (angry receiver, neutral giver) and 31 in the neutral conditions (neutral receiver, neutral giver); 48 dogs were tested in the indirect context and 43 in the direct context.

### Analyses

The primary variable used to determine dogs’ preference for interaction upon release was the dog’s Choice, which could be one of the actors, straight to the table or no approach. We analysed the choice behaviour as both binary (choosing or not making a choice) and nominal (specific choice) data, to test whether valence and food accessibility affected the decision to make a choice and the specific choices made (approach actors, table or other).

In addition, we analysed the gazing behaviour of the dogs in the different conditions, as determined by their head orientation towards a particular person’s upper body, which is potentially a more informative region and thus predicted to be associated with information gathering. We further examined looking (head orientation to any part of the persons present or elsewhere), body orientation (as per looking), position in the room (location relative to particular individuals or specific areas of the room) and sniffing (any part of the scene—see Table 1 for details of all behaviours) using multivariate and univariate analyses of variance. Even though gazing and looking are visual exploration behaviours, they are conceptually and operationally different. Gazing is the looking behaviour towards the upper half of the person’s body, whilst looking refers to the looking behaviour in general, that is, towards any direction. Thus, while gazing could only be directed at the humans present in the experimental area (two actors, owner and experimenter), looking could occur towards humans, the objects and to any empty space.

**Table 1:** Description of the behavioural categories

Category	Description
Approaching	Dog entering a target area (i.e. actors areas, table area). Measured by the presence of the frontal half of the dog’s body in the designated area. Measured in frequency.
Gazing	Eyes directed at the upper body of the actors, owner or experimenter. Measured by the direction of the dog’s head allowing visual access to the target region of the human elements in the scene. Measured in duration.
Looking	Eyes directed at any part of the body of the actors, owner and experimenter, at the table and out. Measured by the direction of the dog’s head. Measured in duration.
Body orientation	Dog’s body directed towards the actors, table, owner, experimenter or out. Measured by an imaginary line created from the point at the base of the dog’s neck, in between its shoulders. Measured in duration.
Positioning in the room	Dog’s presence in a target area (i.e. actors areas, table area, middle area, owner area, experimenter area, back area, out). Measured by the presence of the frontal half of the body of the dog in the designated area. Measured in duration.
Sniffing	Olfactory exploration of targets (i.e. actors, table, owner, experimenter, out), measured by the close proximity of the dog’s nose to a surface and distinct nostrils movement (detected visually and/or acoustically). Measured in duration.
Touching	Physical contact between the frontal area of the dog (e.g. nose, mouth, front paws) and the target (e.g. actors, table, owner). Measured in duration.

The videos obtained from the two digital video cameras were synchronised to allow robust coding of the behaviours and behavioural responses, which was done using frame-by-frame and real time speeds in Solomon Coder Beta (www.solomoncoder.com). We coded the dogs’ first choices as well as the seven exploratory behaviour categories listed in Table 1: approaching, gazing, looking, body orientation, positioning in the room, sniffing and touching.

Overall, we used a non-parametric approach to test choices and approaches (frequency variables) and multivariate and univariate models of analysis of variance to test the other categories (duration variables). The significance level was always 0.05. All analyses were conducted in IBM SPSS Statistics. Touching was a rare event and, therefore, no inferential analysis was conducted for this variable. A second researcher coded all behaviours for 25% of the videos, which were drawn randomly from the total sample of analysed subjects. Both primary and secondary coders were blind to the emotional expressions and experimental conditions. Inter-observer reliability measures yielded very high and significant correlation as well as concordance values. See tables S2 and S3 for details.

For the other categories, we used the relative durations of each variable (e.g. duration of looking at the emotional actor divided by the total duration of looking) in three different approaches to investigate the effect of emotional group and context (direct or indirect) on the responses towards: (1) the emotional actor and the unemotional actor (*N* = 60); (2) others, e.g. the owner and the experimenter (*N* = 91); and (3) the actors combined, regardless of their emotional status in all trials (*N* = 91). All assumptions for univariate and multivariate analysis of variance models were met and only very well adjusted models were used. When necessary, to meet the assumptions of each model, outliers were excluded (see Supplementary Materials for details). Since there were three levels within the factor group (positive, negative and neutral) in models 2 and 3, we used Scheffe post hoc tests to assess where were the differences between the levels of each model.

**Fig. 2:**
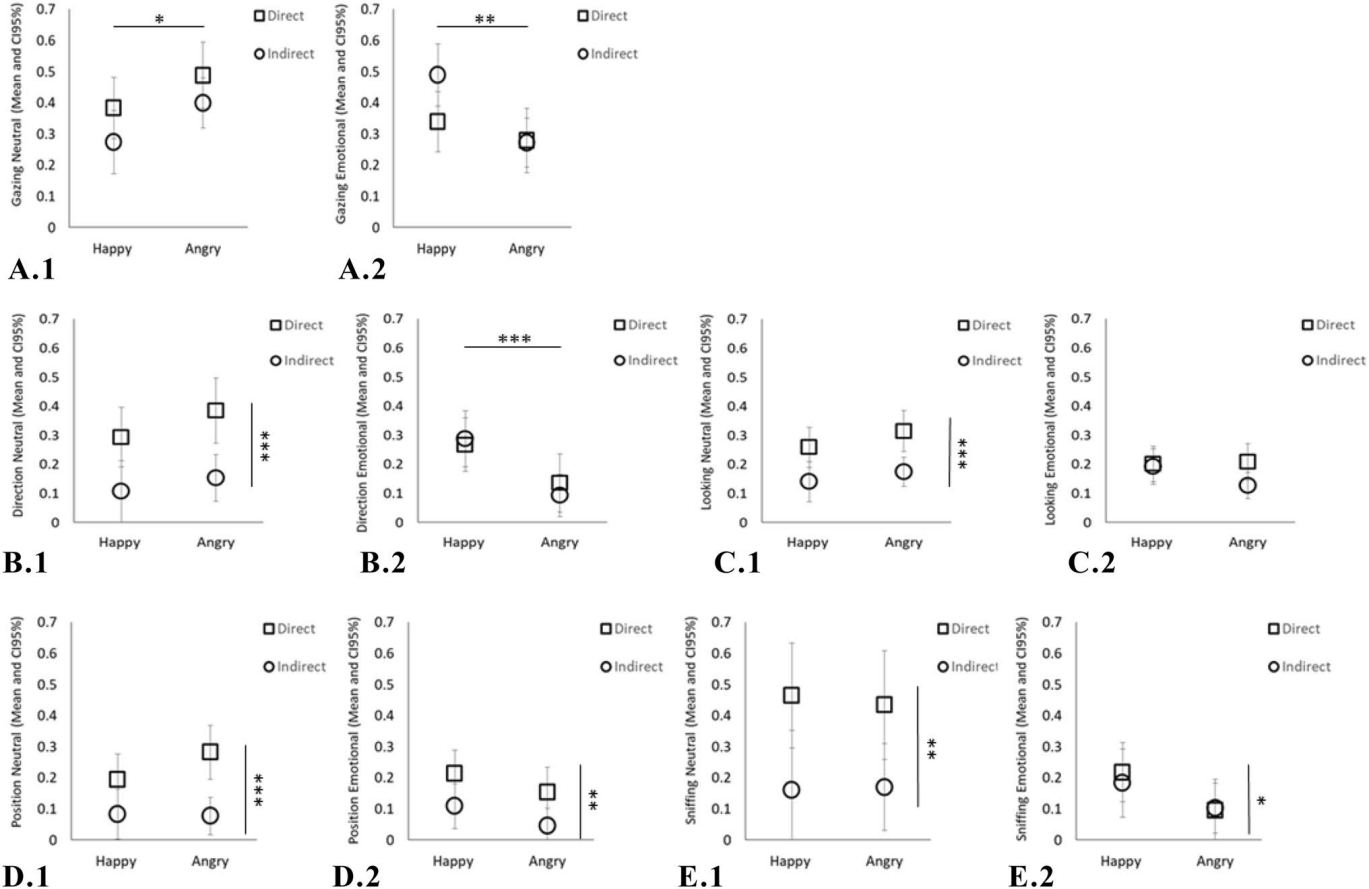
Relationship between dog behaviour towards the different actors (neutral actor—graphs 0.1 or the emotional actor—graphs 0.2), emotional condition (happy or angry) and food accessibility (direct or indirect). Within each graph, the *Y*-axis refers to the behavioural category and the *X*-axis refers to the emotional condition (positive on the left, i.e. happy, and negative on the right, i.e. angry). Results are presented as mean relative duration with 95% confidence interval for gazing (**A**), body orientation—“direction” (**B**), looking (**C**), position in the room—“position” (**D**) and sniffing (**E**). We found no significant interactions between factors. The horizontal and vertical bars indicate where significant effects were found: *p ≤ 0.05; **p ≤ 0.01; ***p ≤ 0.001

We initially focussed on the impact of emotion on choice. Second, we included the emotional group (positive or negative) and food accessibility (direct or indirect access) as fixed factors and the responses directed at the neutral actor (giver) and the emotional actor (receiver) as dependent variables in the model. Because we wanted to compare the responses in relation to positive and negative emotional expressions (see Fig. 2), we did not include the trials when there was no emotion exhibited by either actor.

The third analysis considered the potential effects of emotional group and food accessibility on the responses directed at others (i.e. owner, experimenter, out) and so examined all trials, i.e. positive, negative and neutral trials (*n* = 91).

The fourth analysis examined whether the two factors, “type of exchange between the actors” and “accessibility of food”, affected how dogs behaved towards the two actors who had been involved in the social interaction. We included emotional group and food accessibility as factors and the response directed towards the actors (combined, regardless of being neutral or emotional) as a dependent variable. Accordingly, we analysed the trials from all conditions, i.e. positive, negative and neutral trials (*n* = 91).

As each subject was assigned to only one emotional condition and dogs were tested only once, the data were independent and so subjects were not considered as random factors in the models (see Supplementary Materials for more details).

## Results

### Factors affecting the choice of dogs

Across trials, the frequency of choosing versus not choosing was significantly different between groups. A choice was made in 93% of positive, 65% of neutral and 58% of negative trials. There was no side bias in the dog’s behaviour (Chi-square test, *X*^2^ = 0.89, *p* = 0.64), meaning that the dogs were not responding based on any asymmetry of the room or biased by the side of the owner. Further, there was no bias towards any actor (binomial test, *p* = 0.104).

A binary regression showed that dogs were almost 14 times more likely to choose an actor rather than not make a choice after seeing a positive emotional exchange (*p* = 0.005, 95% CI 2.153–82.068). When a choice was made, we found a discriminative response dependent on emotional valence (Chi-square test, *X*^2^ = 17.58, *p* < 0.0001), with a preference for the positive actor. In the positive conditions, dogs chose the receiver (happy; 68%) more than the giver (neutral; 32%) and, in the negative conditions, the giver (neutral; 95%) more than the receiver (angry; 5%). There was no difference between givers and receivers when both were neutral (Chi-square test, *X*^2^ = 0.2, *p* = 0.65), indicating that the emotional expression of the actors rather than cues concerning their motivational tendency (i.e. being a giver or a receiver) were being used by subjects to inform their choice.

### Impact of emotional expression on dogs

The emotional reaction of the receiver affected gazing and body orientation (Fig. 2; A1, A2, B1 and B2). Dogs gazed longer at the upper body of the happy actor in the positive conditions (*F*_1,60_ = 8.139, *p* = 0.006; Fig. 2, A2) and longer at the upper body of the neutral actor in the negative conditions (*F*_1,60_ = 4.414, *p* = 0.024; Fig. 2, A1). Dogs in the positive condition spent more time oriented towards the emotional actor than dogs in the negative condition (*F*_1,60_ = 12.289, *p* = 0.001; Fig. 2, B2). These results are consistent with their subsequent approach preferences and with the active acquisition of emotional information.

### Impact of food accessibility on dogs

Food accessibility also affected dogs’ decision-making. Dogs were 30 times more likely to make a choice when there was direct access to the food (binary regression, *p* < 0.0001, 95% CI 5.790–146.793). However, overall, food accessibility did not influence (upper body) gaze allocation (*F*_1,91_ = 3.849, *p* = 0.053; Fig. 2, A1 and A2), but dogs looked at (*F*_1,91_ = 31.920, *p* < 0.0001; Fig. 2, C1), oriented their body towards (*F*_1,91_ = 27.738, *p* < 0.0001; Fig. 2, B1), stayed in the area of (*F*_1,91_ = 63.534, *p* < 0.0001; Fig. 2, D1 and D2) and sniffed (*F*_1,91_ = 14.407, *p* < 0.0001; Fig. 2, E1 and E2) the actors more in the direct than indirect conditions, which might be explained by the proximity between the actor and the food.

Furthermore, analyses of the valenced conditions (positive and negative trials only; Fig. 2) revealed that food accessibility again did not affect gazing (*F*_2,60_ = 3.036, *p* = 0.056). However, dogs looked at (*F*_1,60_ = 14.995, *p* < 0.0001), oriented their body towards (*F*_1,60_ = 16.501, *p* < 0.0001), stayed in the area of (*F*_1,60_ = 15.661, *p* < 0.0001) and sniffed (*F*_1,60_ = 10.769, *p* = 0.002) the neutral actor (giver) more in the direct compared to the indirect conditions regardless of the valence of the receiver (happy or angry), indicating a preference for obtaining the resource from the person who was not emotionally-expressive.

## Discussion

In the conditions when dogs could not reach the food by themselves, the valence of the displays played an important and informative role. When dogs had to use the human as a social tool, they showed more discriminative behaviour. These results show that dogs actively acquire information from affective cues produced by people and make functional use of this to solve ecologically relevant problems. To do this, they must be able to infer the emotional states of people from representations they have generated and stored in their memory, probably based on their previous experience with each type of stimulus. To our knowledge, this ability has only previously been demonstrated in humans. Here, we show that it occurs in dogs even when the information is only available from the passive observation of third-party interactions with emotional reactions that are triggered by a neutral object and are not directed towards the dog. During testing, the dogs had to infer the potential consequences of the emotional exchange they had witnessed previously and make a choice based on the information they had active in their memory about the actors and the different aspects of the social context. Thus, we not only provide evidence that the capacity to infer emotional states and consequences of specific emotional expressions is not unique to humans; but that it is also present in an animal that is phylogenetically distant from humans.

We also found that dogs’ choice was not affected by the action (or motivation) of the demonstrators (i.e. giving or receiving). Dogs relied on the emotional information, even though they can perceive humans as potential helpers for solving problems (Horn et al. 2011) and can develop preferences for people that act cooperatively (Chijiwa et al. 2015; Marshall-Pescini et al. 2011)*.* We found no difference in the likelihood of dogs choosing the giver, who could be understood as a cooperative party, and the receiver, meaning they were acquiring relevant information about the emotional displays.

To infer the consequences of emotions requires an individual to establish causal and temporal relationships between discrete events, i.e. the emotional expression and the potential subsequent action (Leon-Rodriguez et al. 2008). Knowledge about the potential behavioural outcomes of others’ emotional experiences is crucial for making appropriate choices, reacting adequately and executing more effective social actions. Humans use social information to interact with others based on their experience, not only from their previous direct interactions but also from observing the interactions between other individuals (van Kleef et al. 2009). Humans often observe others’ emotional expressions and use the information to guide their own course of action. Our results demonstrate that dogs can actively acquire relevant information from affective displays, match these with information about emotional expressions and consequences, and use these associations to predict the potential outcome of the emotional state of others in their current decision-making.

Chimpanzees (Buttelmann et al. 2009), capuchin monkeys (Morimoto and Fujita 2012) and domestic dogs (Buttelmann and Tomasello 2013; Ford et al. 2019) approach or avoid novel stimuli according to the valence of others’ expressions. However, perceptually-based social information mechanisms, such as social referencing and categorisation, refer to the use of predictive cues to guide behaviour. They can be due, for example, to the association of specific facial configurations with rewards, and do not necessarily require knowledge of the content of the stimulus. They do not imply the actual acquisition of the emotional information or the inference about the emotional state of another individual (Leon-Rodriguez 2008)*.* Waller and colleagues (2016) showed that a captive crested macaque could learn to associate the exhibition of facial expression of conspecifics to social outcomes. Even though this indicates a highly adaptive feature, the subject could be responding to perceptual associations between specific facial configurations and social configurations without the involvement of inferential processes.

Several animal species are sensitive to emotions, with evidence that, besides primates, dogs and horses not only discriminate emotional expressions but truly recognise them, by extracting emotional information from faces and vocalisations and integrating this information into a robust percept (Albuquerque et al. 2016; Nakamura et al. 2018) and thus have representations of, at least, main emotional categories (e.g. positive and negative). The more widespread presence of this ability might be due to its highly adaptive value, especially towards mediating affiliative behaviours, avoiding harmful interactions and, thus, increasing survival chances (Ferretti and Papaleo 2018). During their lifetime, animals gather information from their experience with objects, individuals, events and social interactions and store this information in their memory (Ades 1993), which will be used when discriminating, categorising and recognising different aspects of their physical and social environment. This allows the generation of embodied representations that will be expressed by differential muscle movements, changes in body posture and facial expressions (Farb et al. 2013).

In addition to modulating sensory inputs, emotional expressions can reflect emotional states. Indeed, humans acknowledge the meaning of emotional expressions, processing information that goes beyond the perceptual level (Adolphs 2002). This information is not present in the structure of the emotional expression itself; it is retrieved from the person’s past experiences with the world. For example, seeing happiness might activate these embodied representations and trigger approaching behaviours from the observer towards the signaller (Hawk et al. 2012) whilst anger expressions, perceived as a potential threat (Smith et al. 2016), might facilitate avoidance-related behaviours (Marsh et al. 2005). Indeed, a recent study using horses has shown that non-human animals can remember the emotional cues displayed by another individual (Proops et al. 2018). Remembering affective interactions greatly benefits the social relationships established between individuals, for either affiliative, agonistic or avoidant behaviours.

In this study, we used complementary measures to test our hypotheses. In addition to revealing that dogs choose more the happy actors and were more likely to make a choice in the positive conditions, we found that gazing was a crucial variable, providing us with information of dogs’ visual exploration of the area of the body from where most of the emotional information could be obtained. According to a few studies on dog–human communication (Cavalli et al. 2020; Savalli et al. 2016; Somppi et al. 2016), the gazing behaviour allows the acquisition of relevant social information from humans. It is discussed that dogs’ gazing behaviour in the dog–human relationship is responsible for complex processes including hormone-mediated mechanisms that facilitate bonding and have social rewarding effects (Nagasawa et al. 2015). Interestingly, the effect of the emotional cues on gazing was only found for the behaviours directed to the actors, i.e. there was no significant effect regarding gazing at the owner, at the experimenter or at the actors combined. This might suggest that gazing was closely related to the acquisition of information, since the effects were only found when taking into account the emotional exchange and the affective quality of the interaction. Also, we found no effect of type of access (direct or indirect access to the food) on gazing, which means that the occurrence of this behaviour was not linked to the availability of food. We suggest this may be considered to be a way of obtaining relevant information about the social environment.

The effect of emotional valence on body position could be seen as a carryover effect from gazing. However, our data does not support this claim. First, there is no strict physical relationship between body position and gazing, as a dog can be directed to one side and gaze at another. Second, we found no significant effect for looking, a variable that accounted for the visual allocation of the subjects to any part of someone’s body as well as the rest of the scene. Therefore, our results suggest that dogs were actively obtaining information from the actors. Surprisingly, while the effects of the emotional group were very clear for gazing and less clear for body position, they were non-existent for the other duration variables, suggesting that these other behaviours were not important to the processing of the emotional information.

Gaze allocation towards the upper half of the actors’ body was consistently affected by the valence of the actors’ displays. This pattern was revealed only for the test (emotionally charged) trials, where there was an actual exhibition of emotional information, and this is consistent with the recent finding of Correia-Caeiro et al. (2021) that dogs attend more to the body when viewing emotional displays by people. However, type of access did not influence gaze allocation in the current study. Thus, the emotionality of the context per se, rather than a specific emotional display, affected actor-directed gazing, a behaviour associated with the acquisition of social information from humans (e.g. Savalli et al. 2014). However, gazing at others (i.e. the owner and the experimenter (neutral) or the actors combined—regardless of valence) was not affected in this way. This supports the conclusion that gazing at the upper body is related to the active acquisition of emotionally salient information.

Dogs responded selectively to the social information on the basis of the wider context and did not use the affective information from humans’ visual displays indiscriminately. When they could access the food by themselves they showed less differential behavioural responses. This is likely to be because they did not need to use the humans as a social instrument to obtain the desired resource and because the available food was a more salient stimulus, driving their attention allocation.

The behavioural differences observed regarding food accessibility are consistent with (a) free access to the food in the direct conditions resulting in greater interest in the food close to the actors; and (b) the need to use humans to obtain the resource in the indirect conditions. What is of particular importance to appreciate is that when the food is unreachable, the potential significance of the emotional displays of the actors is greater, since there is a dependence on others to achieve a desired goal. It is likely that flexible social strategies have evolved in dogs as a species as well as during each individual’s ontogenetic pathways, allowing the development of refined social mechanisms. In this sense, the ability to acquire information from human affective cues and to make functional use of them, i.e. emotionally charged information-based decision-making, might be of importance to the success of dogs in the anthropogenic environment. Here, we show that these capacities are biologically and psychologically relevant to non-human animals and might be much more widespread than previously thought. The ability to actively acquire information from emotional expressions and use this information to adjust behaviour and decide with whom to interact is clearly highly advantageous to a species that lives in complex social environments. Heterospecific emotion recognition in non-human animals allows individuals to mediate affiliative behaviours and avoid potentially harmful interactions, thus increasing fitness (Ferretti and Papaleo 2018; Farb et al. 2013). Additionally, the knowledge of the potential behavioural outcomes of others’ emotional expressions is of great adaptive value as it is likely to play a crucial role in optimal decision-making, thus allowing more effective social action.

The spontaneous, free and social-ecologically relevant nature of the task and the nuances of the conditions, such as contrasting different types of contexts and presenting different combinations of emotional expressions in a naturalistic and indirect manner, allow us to make robust conclusions on dogs’ ability to infer implicit information from humans and use it in a functional way. We believe the current study provides important theoretical and applied contributions to research on emotion perception, affective communication and dog cognition. Future studies exploring dogs’ sex, breed, age and length of time living with the owners, as well as assessing other species, looking at other emotional expressions and exploring other sensory modalities, will build on to these findings and allow a deeper understanding of the underlying mechanisms of emotion recognition and inference in animals.

## Supplementary Information

Below is the link to the electronic supplementary material.

Supplementary file1 (DOCX 1306 kb)

Supplementary file2 (MP4 27582 kb)

Supplementary file3 (MP4 25980 kb)

Supplementary file4 (MP4 24707 kb)

## Data Availability

All data and materials will be made fully available upon acceptance of the manuscript.
