# Supplementary material for: Dogs can infer implicit information from human emotional expressions
Source: Anim Cogn. 2021 Aug 14;25(2):231–40. doi: 10.1007/s10071-021-01544-x (PMC8940826; doi:10.1007/s10071-021-01544-x)
Supplement: Supplementary file 1 — Supplementary file1 (DOCX 1306 kb) [file 10071_2021_1544_MOESM1_ESM.docx]

**ANIMAL COGNITION**

**SUPPLEMENTARY INFORMATION**

**Dogs can infer implicit information from human emotional expressions**

Natalia Albuquerque¹^,^²*, Daniel S Mills², Kun Guo³, Anna Wilkinson², Briseida Resende¹

^1^Institute of Psychology, University of São Paulo, São Paulo, Brazil.

^2^School of Life Sciences, University of Lincoln, Lincoln, United Kingdom.

^3^School of Psychology, University of Lincoln, Lincoln, United Kingdom.

*Correspondence to: [nsalbuquerque@usp.br](mailto:nsalbuquerque@usp.br)

ORCID NATALIA ALBUQUERQUE: 0000-0002-0925-0650

ORCID DANIEL S MILLS: 0000-0002-4765-9625

ORCID KUN GUO: 0000-0001-6765-1957

ORCID ANNA WILKINSON: 0000-0002-4500-0181

ORCID BRISEIDA RESENDE: 0000-0001-5932-0189

Experimental Procedure

The experimental session was divided into four parts: 1) habituation; 2) pre-test; 3) observation phase; 4) response phase. Habituation consisted of exposure to the experimental room (owner and experimenter were present but the food bowls were empty. This lasted between five and ten minutes and provided dogs with some time to explore the room and become habituated to it. Instructions regarding the upcoming phases were given to the owner by the experimenter during this time. However, owners were not aware of the specific aims of the project or of the experimental condition until the end of the experimental sessions. Owners did not interact with the table, the stools or the bowls at all. Once habituation was finished, the owner left the room with the dog (always on a leash) and stayed in a previously designated area for a couple of minutes, while the two actors entered the room (without being seen by the dog or the owner) and positioned themselves in front of the stools. The helper baited the bowls and started the cameras. The experimenter reminded the owners of the instructions. In the pre-test phase, owner and dog (on leash) walked back into the experimental room and went towards the table. Owners were asked to ignore the actors completely and the actors were trained to always look ahead and never interact with or respond to the dog or to the owner in any way. Upon reaching the table, the owner took one of the baited bowls and showed it to the dog so the dog could see (and smell) the treats inside. As soon as the dog showed interest in the food, the owner put the bowl back in its initial position on the table and did the same thing with the other bowl. Immediately after this, the owner moved to her/his assigned place in the room and ensured the dog was in the correct place. The dog was positioned at a distance of two meters from the table and the interaction.

Once the subject was set, owners looked at a subtle mark on the wall behind the actors (placed directly in between them) and from this point on never interacted with or responded to the dog or to the actors in any way. Once the dog and owner were in place, the observation phase started (described above). This was followed by the response phase. After the two actors finished the display, sat down and had neutral expressions (Figure S2), the experimenter gave the command to the owner to unclip the leash, thus allowing the dogs to move freely. Neither owner, experimenter or actors interacted with or responded to the dog at all. The behaviour of the dog was recorded during pre-test, observation phase and during the 30 second response phase by the two cameras, one positioned at the back of the room on top of a tall tripod and one under the table on top of a short tripod. The images obtained were complementary and were synchronised for coding. Each dog was tested only once to control for habituation to testing and learning effects and guarantee independence of the data.

Data analysis

*Choice. Choice* was measured as the subject’s first approach (i.e. having the anterior half of the body inside of the target area, see Table S2 for details) and it was analysed as both a nominal and binary variable. The first question we wanted to address was whether there was a difference in choice among the conditions where emotion was exhibited, i.e. the positive and negative conditions (N=60). Therefore, we used chi-square and partitioned chi-square tests to look at the responses towards the emotional actor, the unemotional actor, the table and the likelihood of making no choice (i.e. dogs did not enter either the actors’ or the table area). Moreover, we also used chi-square and partitioned chi-square tests to investigate whether dogs were responding to the emotional content of the interaction instead of other spurious factors. Thus, we analysed the neutral trials (N=31) by the action (giving actor or receiving actor) and all trials (N=91) by the side (left or right) of the demonstrators. The second strategy was to use a binary regression model to investigate the effects of emotional group (positive, negative or neutral) and food accessibility (direct or indirect) on the likelihood of dogs making a choice (i.e. approaching one or the other actor) – in contrast to not choosing at all (i.e. going straight to the table or not entering the target areas). Afterwards, we ran chi-square tests to allow comparisons within the main factors. All assumptions for a binary logistic regression model were met. Finally, we used a binomial test to investigate potential biases toward one of the actor (by their identity).

*Approaches*. We found that *approaching the table* was a rare event (median=0) across all trials (N=91), therefore we did not make inferential analyses of this variable. We used Mann-Whitney for independent samples tests to investigate potential differences between the positive and the negative conditions and the indirect and direct contexts in terms of *approaching the neutral actor* and *approaching the emotional actor* (N=91).

*Gazing*. The first model was a MANOVA including *gazing at the neutral actor* and *gazing at the emotional actor* as dependent variables and group (positive and negative), accessibility of food (direct and indirect) and their interaction as independent variables (N=60, emotional trials). There were no outliers (final model with 60 subjects). *Gazing at experimenter* had too many outliers (median too close to zero) and, thus, was not analysed. The second model was an ANOVA including *gazing at owner* as dependent variable and group (positive, negative and neutral), accessibility (direct and indirect) and their interaction as independent variables (N=91). Five outliers (dog 65, dog 40, dog 33, dog 22 and dog 12) were eliminated from the final model (N=86). The third was an ANOVA including *gazing at actors* as dependent variable and group (positive, negative and neutral), accessibility (direct and indirect) and their interaction as independent variables (N=91). No outliers were excluded.

*Looking*. The first model was a MANOVA including *looking at the neutral actor* and *looking at the emotional actor* as dependent variables and group, accessibility and their interaction as independent variables (N=60; emotional trials). Four outliers (dog 101, dog 55, dog 9 and dog 2) were eliminated from the final model (N=56). The second was a MANOVA including *looking at owner*, *looking at experimenter* and *looking out* as dependent variables and group, accessibility and their interaction as independent variables (N=91, all trials). Five outliers (dog 114, dog 86, dog 75, dog 33 and dog 12) were eliminated from the final model (N=86). The third was an ANOVA including *looking at the actors* as dependent variable and group, accessibility and their interaction as independent variables (N=91). No outliers had to be excluded.

*Body orientation*. The first model was a MANOVA including *body oriented to the neutral actor* and *body oriented to the emotional actor* as dependent variables and group, accessibility and their interaction as independent variables (N=60, emotional trials). Three outliers (dog 101, dog 59 and dog 55) were removed from the final model (N=57).

*Body oriented to the experimenter* was a rare event (median=0) and *body oriented to the owner* and *body oriented to the table* had too many outliers (distribution too far from normal), therefore no inferential analyses were conducted. The second model, thus, was an ANOVA including *body oriented out* as dependent variable and group, accessibility and their interaction as independent variables (N=91, all trials). Three outliers (dog 41, dog 33, dog 12) were eliminated from the final model (N=88). The third was an ANOVA including *body oriented to the actors* as dependent variable and group, accessibility and their interaction as independent variables (N=91). No outliers had to be excluded.

*Position in the room*. The first model was a MANOVA including *position in the neutral actor area* and *position in the emotional actor area* as dependent variables and group, accessibility and their interaction as independent variables (N=60). Six outliers (dog 101, dog 61, dog 55, dog 21, dog 9, dog 2) were eliminated from the final model (N=54). *Position in the experimenter area* and *position in the back area* were rare events (median=0). *Position in the owner area*, *position in the table area* and *position in the middle* showed too many outliers (distribution too far from normal). No analysis was conducted for these variables. The last model was an ANOVA including *position in the actors areas* as dependent variable and group, accessibility and their interaction as independent variables (N=91). No outliers had to be eliminated.

*Sniffing*. The first model was a MANOVA including *sniffing the neutral actor* and *sniffing the emotional actor* as dependent variables and group, accessibility and their interaction as independent variables. Six outliers (dog 109, dog 45, dog 27, dog 17, dog 9 and dog 2) were eliminated from the final model (N=54). *Sniffing owner*, *sniffing experimenter* and *sniffing table* were rare events (median=0). *Sniffing out* showed too many zeros (median very close to zero) and a distribution far from normal. No analysis was conducted for these variables. The last model was an ANOVA including *sniffing the actors* as dependent variable and group, accessibility and their interaction as independent variables (N=91). No outliers had to be eliminated.

***
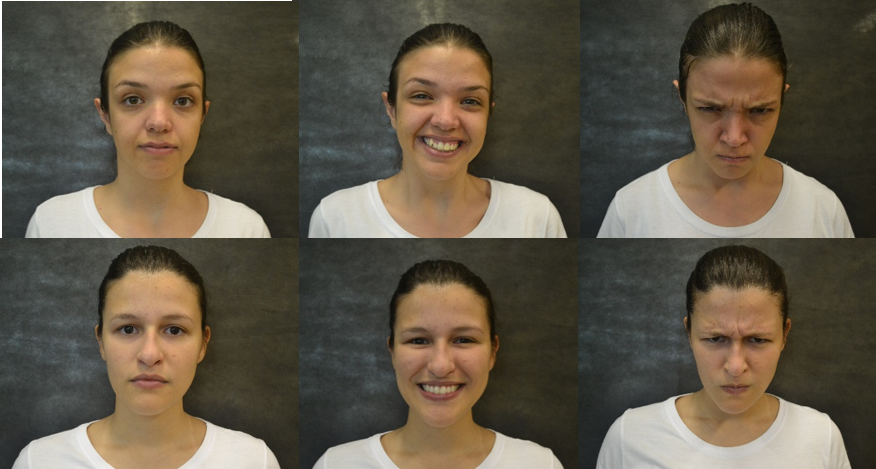
***

**Fig. S1.** Emotional expressions used in the experiment. From left to right: neutral, happy, angry. Stimuli combinations consisted of one neutral actor and another actor that could behave positively, negatively or neutrally depending on the designated emotional group.


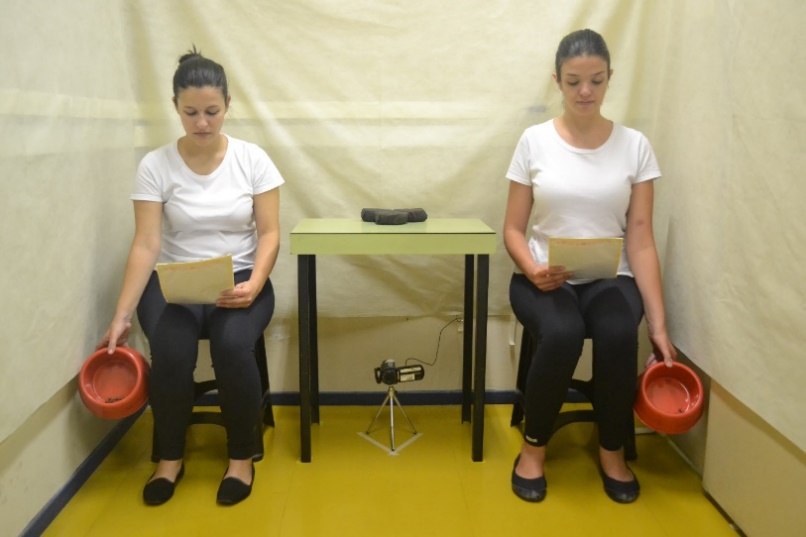

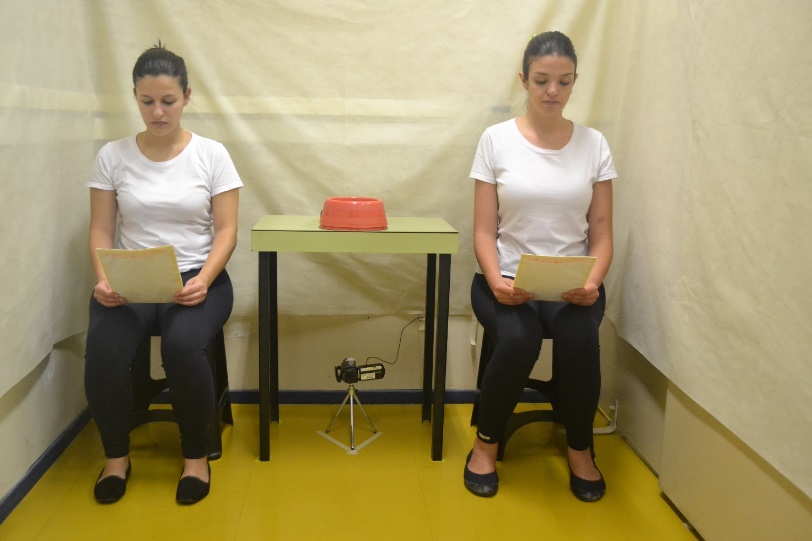


**Fig. S2.** Position of the actors after the emotional interaction (observation phase) and during the response phase. From left to right: direct access to the food (direct condition) and indirect access to the food (indirect condition).


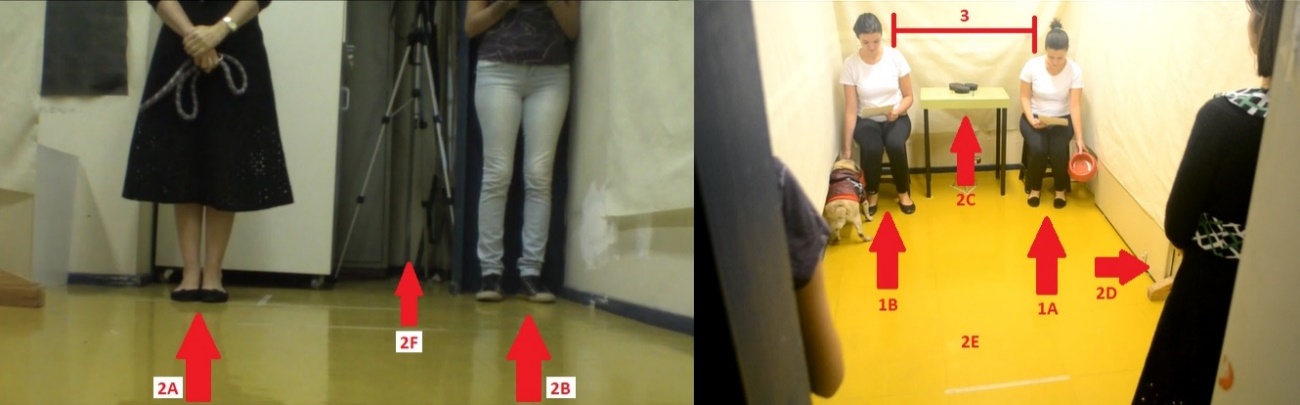


**Fig. S3.** Variables measured within each approach of analysis: 1) responses towards the neutral actor and the emotional actor separately (N=60); 2) responses towards the other elements (N=91); 3) responses towards the actors combined, regardless of the emotional expression (N=91). 1A and 1B represent the behaviours concerning the neutral and the emotional actor. 2A, 2B, 2C, 2D, 2E and 2F represent the behaviours concerning the owner, the experimenter, the table, out, the middle and back area, respectively. 3 represents the behaviours concerning the actors combined.

**Table S1.** Information of the dogs tested.

| **Subject** | **Dog** | **Breed** | **Age (years)** | **Sex** | **Status** | **Analysed** |
| --- | --- | --- | --- | --- | --- | --- |
| 1 | Bartolomeu | Pug | 2 | Male | neutered | yes |
| 2 | Caruso | Pug | 6 | Male | neutered | yes |
| 3 | Calabresa | Pug | 4 | Female | neutered | yes |
| 4 | Chérie | Stray | 3 | Female | neutered | yes |
| 5 | Pistache | Stray | 3 | Male | neutered | no |
| 6 | Preta | Stray | 11 | Female | neutered | yes |
| 7 | Maitê | Shetland Sheepdog | 0.8 | Female | entire | yes |
| 8 | Thor | Shetland Sheepdog | 1.6 | Male | neutered | no |
| 9 | Iara | Stray | 2 | Female | neutered | yes |
| 10 | Naná | Bernese | 5 | Female | neutered | yes |
| 11 | Willow | Bernese | 7 | Female | neutered | yes |
| 12 | Chico | Stray | 2 | Male | neutered | yes |
| 13 | Astana | Springer Spaniel | 3 | Female | neutered | yes |
| 14 | Capitu | Stray | 6 | Female | neutered | no |
| 15 | Duff | Shih Tzu | 1.6 | Male | neutered | yes |
| 16 | Thor | Shih Tzu | 1.6 | Male | neutered | no |
| 17 | Lunna | Stray | 6.5 | Female | neutered | yes |
| 18 | Juju | Stray | 2 | Female | neutered | yes |
| 19 | Pérola | Golden Retriever | 3.7 | Female | neutered | no |
| 20 | Cafu | Stray | 6 | Male | neutered | no |
| 21 | Maya | W. H. White Terrier | 1.3 | Female | neutered | yes |
| 22 | Penélope | Stray | 2.5 | Female | neutered | yes |
| 23 | Tora | Staffordshire Bull Terrier | 1.5 | Female | neutered | yes |
| 24 | Zapata | Border Collie | 2.5 | Male | neutered | yes |
| 25 | Adele V. | Papillon | 3.5 | Female | neutered | yes |
| 26 | Zattar | Stray | 4 | Male | neutered | yes |
| 27 | Julie | Lhasa Apso Mix | 2.5 | Female | neutered | yes |
| 28 | Nix | Stray | 5 | Female | neutered | yes |
| 29 | Pudim | Poodle | 3 | Male | neutered | yes |
| 30 | Link | Stray | 5 | Male | neutered | yes |
| 31 | Colie | Stray | 4 | Female | neutered | yes |
| 32 | Juca | Bichon Frisé | 10.5 | Male | neutered | no |
| 33 | Lolla | Stray | 3 | Female | neutered | yes |
| 34 | Kevin | Shetland Sheepdog | 4.1 | Male | neutered | yes |
| 35 | Brida | Jack Russel | 1.8 | Female | neutered | no |
| 36 | Kyara | Lhasa Apso | 4 | Female | neutered | no |
| 37 | Tutty | Shih Tzu | 5 | Male | neutered | no |
| 38 | Cacau | Stray | 5 | Female | neutered | yes |
| 39 | Chucrute | Boxer | 7 | Male | entire | no |
| 40 | Bolota | Shih Tzu | 2.1 | Male | entire | yes |
| 41 | Thor | Golden Retriever | 5 | Male | neutered | yes |
| 42 | Madona | Rottweiler | 5.8 | Female | neutered | yes |
| 43 | Vênus | Doberman | 5.9 | Female | neutered | no |
| 44 | Chincha | Yorkshire | 5 | Male | neutered | yes |
| 45 | Estrelinha | Stray | 2.5 | Female | neutered | yes |
| 46 | Winnie | Stray | 12 | Female | neutered | yes |
| 47 | Dazs | Stray | 4 | Female | neutered | yes |
| 48 | Mel | Golden Retriever | 4 | Female | neutered | yes |
| 49 | Nina | Shetland Sheepdog | 3.3 | Female | neutered | yes |
| 50 | Lisa | Dachshund | 7 | Female | neutered | no |
| 51 | Spyke | Dachshund | 5 | Male | entire | yes |
| 52 | Nega | Stray | 3.5 | Female | neutered | yes |
| 53 | Luna | Lhasa Apso | 3 | Female | entire | yes |
| 54 | Milka | Shih Tzu | 2.3 | Female | neutered | yes |
| 55 | Bolota B. | Pug | 9 | Male | neutered | yes |
| 56 | Axel | Samoieda | 6 | Male | neutered | no |
| 57 | Polly | Stray | 6 | Female | neutered | yes |
| 58 | Onur | Golden Retriever | 2.1 | Male | neutered | no |
| 59 | Guta | Schnauzer | 5.5 | Female | neutered | yes |
| 60 | Lua C. | Stray | 4 | Female | neutered | yes |
| 61 | Maria Q. | Giant Schnauzer | 1.5 | Female | neutered | yes |
| 62 | Layla | Golden Retriever | 8 | Female | neutered | yes |
| 63 | Capitu | Cocker Spaniel | 5 | Female | neutered | yes |
| 64 | Mel | Stray | 4 | Female | neutered | no |
| 65 | Maria Q. | Stray | 7 | Female | neutered | yes |
| 66 | João | Stray | 7 | Male | neutered | yes |
| 67 | Miski | W. H. White Terrier | 10 | Female | neutered | yes |
| 68 | Melrose | W. H. White Terrier | 10 | Female | neutered | yes |
| 69 | Vivi Jr. | W. H. White Terrier | 12 | Female | neutered | yes |
| 70 | Laika | Stray | 3 | Female | neutered | yes |
| 71 | Grappa | Labrador+Golden Mix | 6 | Female | neutered | yes |
| 72 | Google | Golden Retrievier | 7 | Male | neutered | yes |
| 73 | Mambo | Golden Retrievier | 6 | Male | neutered | yes |
| 74 | Luke S. | Pug | 3.7 | Male | neutered | yes |
| 75 | Miky | Shih Tzu | 6 | Male | neutered | yes |
| 76 | Patrícia | Yorkshire | 10 | Female | neutered | yes |
| 77 | Peter Parker | Yorkshire | 10 | Male | neutered | no |
| 78 | Tabata | Yorkshire | 5 | Female | entire | yes |
| 79 | Maria José | Yorkshire | 5 | Female | entire | yes |
| 80 | Maria Lúcia | Yorkshire | 5 | Female | entire | yes |
| 81 | Cacau | Basset Hound | 1.9 | Male | entire | yes |
| 82 | Luna | Basset Hound | 3.9 | Female | neutered | yes |
| 83 | Vitória | Stray | 11 | Female | neutered | yes |
| 84 | Penélope | Stray | 6.8 | Female | neutered | yes |
| 85 | Lord Zé | Pug | 9 | Male | neutered | yes |
| 86 | João | Yorkshire | 7 | Male | neutered | yes |
| 87 | Sofie | W. H. White Terrier | 8 | Female | neutered | no |
| 88 | Astato | Stray | 5 | Male | neutered | yes |
| 89 | Duquesa | Stray | 3 | Female | neutered | yes |
| 90 | Snoopy | Dachshund | 3 | Male | neutered | yes |
| 91 | Jobim | Jack Russel | 3 | Male | entire | yes |
| 92 | Lili | Poodle Mix | 8.5 | Female | neutered | yes |
| 93 | Toddy | Welsh Corgi | 4 | Male | neutered | yes |
| 94 | Simba | Stray | 2 | Male | neutered | yes |
| 95 | Mel | Stray | 7 | Female | neutered | yes |
| 96 | Toti | Lhasa Apso | 7 | Male | neutered | yes |
| 97 | Sol | Stray | 4 | Female | neutered | no |
| 98 | Noah | Golden Retriever | 2.5 | Male | neutered | yes |
| 99 | Ana | Stray | 5 | Female | entire | yes |
| 100 | Pingo | Stray | 6 | Male | neutered | yes |
| 101 | Bali | Border Collie | 7 | Male | entire | yes |
| 102 | Sophia | Stray | 6.5 | Female | neutered | no |
| 103 | Filipa | French Bulldog | 3 | Female | neutered | yes |
| 104 | Apolo | Beagle | 3 | Male | neutered | yes |
| 105 | Marie | Stray | 2.3 | Female | neutered | no |
| 106 | Frida | Stray | 2.3 | Female | neutered | yes |
| 107 | Gummy | Pinscher | 9 | Female | neutered | no |
| 108 | Angel | Stray | 7.5 | Female | neutered | yes |
| 109 | Doug | Poodle | 4 | Male | neutered | yes |
| 110 | Bela | Poodle Mix | 1.5 | Female | neutered | yes |
| 111 | Wolverine | Shih Tzu | 1.3 | Male | entire | yes |
| 112 | Zyon | Shih Tzu | 2.5 | Male | entire | yes |
| 113 | Jack | Stray | 3 | Male | neutered | no |
| 114 | Lady | Golden Retriever | 6 | Female | neutered | yes |

**Table S2.** Values of the correlation and concordance tests run for the double coding data with respect to the analysed frequency variables.

|  | **Spearman's correlation** | | **Kendall's concordance** | |
| --- | --- | --- | --- | --- |
| **Variable** | ***R*** | ***p*-value** | ***W*** | ***p*-value** |
| Choice | 1.000 | <0.0001 | 1.000 | <0.0001 |
| Approach actor A | 0.946 | <0.0001 | 0.940 | <0.0001 |
| Approach actor B | 0.944 | <0.0001 | 0.923 | <0.0001 |

**Table S3.** Values of the correlation and concordance tests run for the double coding data with respect to the analysed duration variables.

|  | **Pearson's correlation** | | **Kendall's concordance** | |
| --- | --- | --- | --- | --- |
| **Variable** | ***r*** | ***p*-value** | ***W*** | ***p*-value** |
| Look actor A | 0.985 | <0.0001 | 0.906 | <0.0001 |
| Look actor B | 0.983 | <0.0001 | 0.948 | <0.0001 |
| Look Owner | 0.989 | <0.0001 | 0.949 | <0.0001 |
| Look Experimenter | 0.936 | <0.0001 | 0.810 | <0.0001 |
| Look Out | 0.994 | <0.0001 | 0.958 | <0.0001 |
| Body actor A | 0.989 | <0.0001 | 0.924 | <0.0001 |
| Body actor B | 0.970 | <0.0001 | 0.930 | <0.0001 |
| Body Out | 0.980 | <0.0001 | 0.897 | <0.0001 |
| Position actor A | 0.997 | <0.0001 | 0.981 | <0.0001 |
| Position actor B | 0.991 | <0.0001 | 0.934 | <0.0001 |
| Gaze actor A | 0.962 | <0.0001 | 0.921 | <0.0001 |
| Gaze actor B | 0.936 | <0.0001 | 0.874 | <0.0001 |
| Gaze Owner | 0.983 | <0.0001 | 0.864 | <0.0001 |
| Sniff actor A | 0.996 | <0.0001 | 0.924 | <0.0001 |
| Sniff actor B | 0.969 | <0.0001 | 0.881 | <0.0001 |

Video S1.

Example of Neutral trial

Video S2.

Example of Positive trial

Video S3.

Example of Negative trial
